# Supplementary figures and images for: Development of Anti‐Inflammatory Extracellular Vesicles by Surface Expression of Syndecan‐4
Source: J Extracell Vesicles. 2026 Apr 2;15(4):e70266. doi: 10.1002/jev2.70266 (PMC13045914; doi:10.1002/jev2.70266)

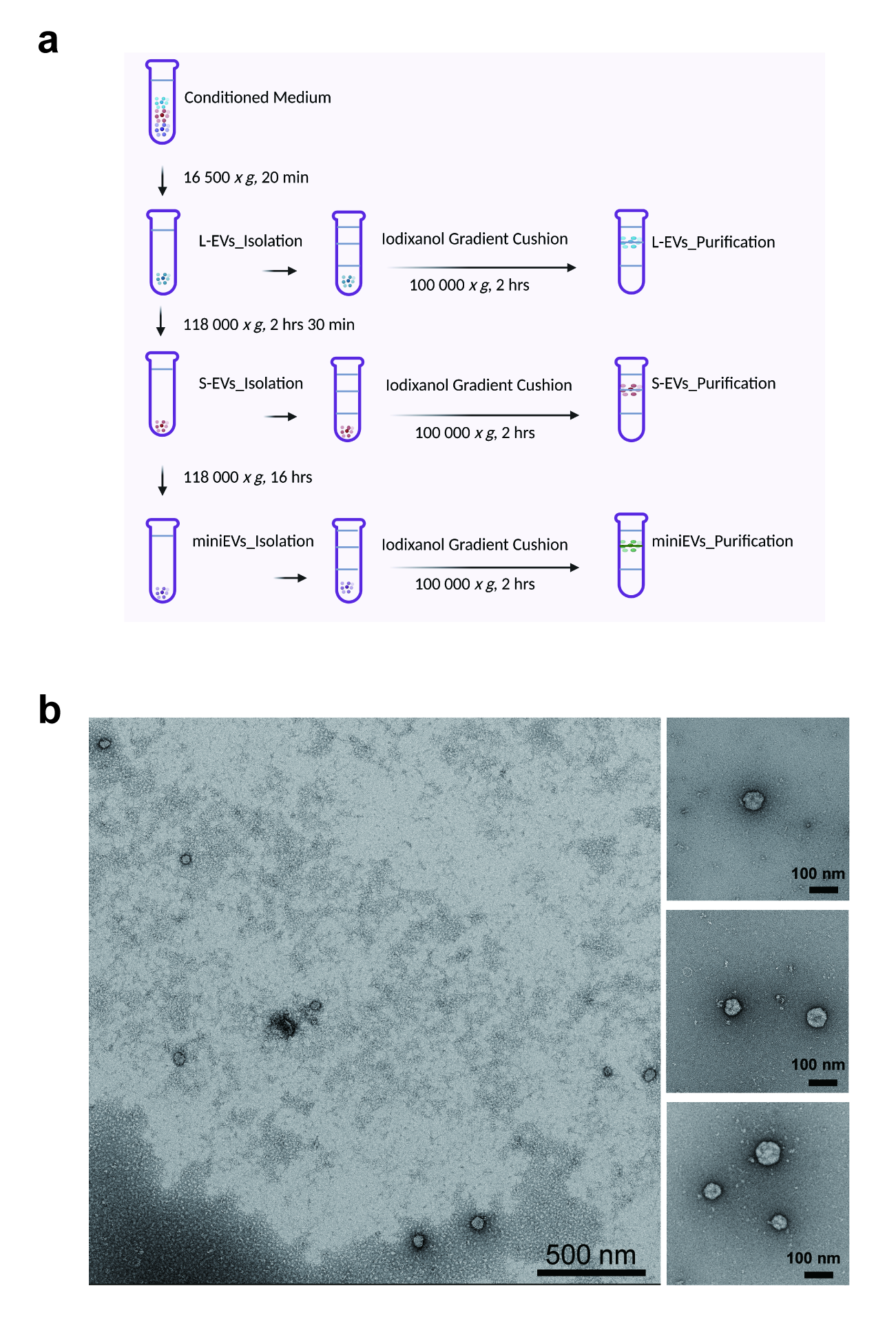

Supplement: Supplementary file 1 — Supplementary Figure 1. Method for enrichment of L‐EVs, S‐EVs, and miniEVs from conditioned medium from HEK293F cells. a) The centrifugation methods for the enrichment of the different EVs. b) TEM images of HEK293F miniEVs at different magnification. Bigger image: 22,000 X magnification; Smaller images: 73, 000 X magnification. [file JEV2-15-e70266-s002.tif]

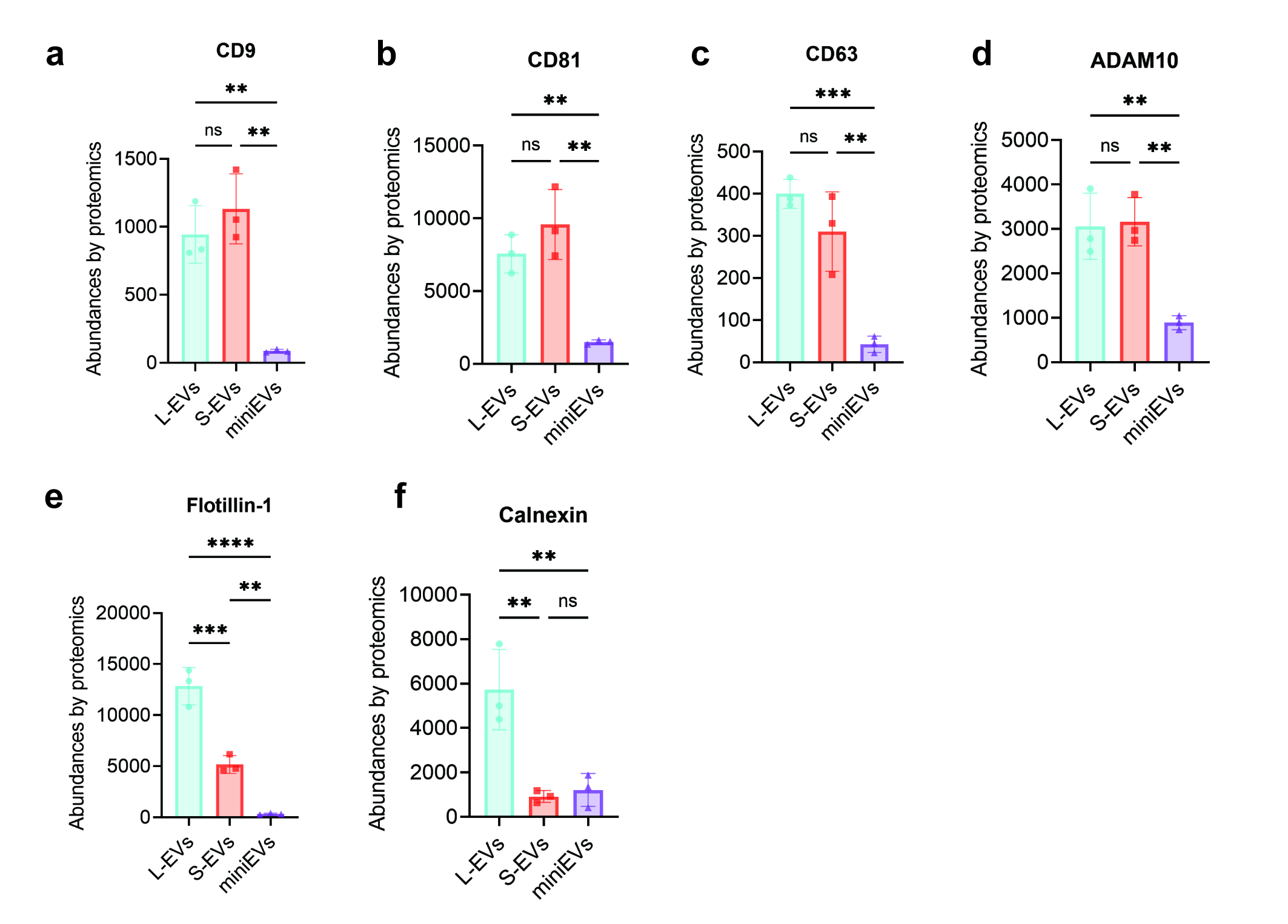

Supplement: Supplementary file 2 — Supplementary Figure 2. Protein expression of EV proteins in the proteomic analysis of the HEK293F EVs. a‐e) The abundance in the LC‐MS/MS analysis for the commonly analyzed EV proteins; CD9 (a), CD81 (b), CD63 (c), ADAM10 (d), and Flotillin‐1 (e). f) The abundance in the LC‐MS/MS analysis for the endoplasmic reticulum protein Calnexin. Data were analyzed using one‐way ANOVA. **, P < 0.01; ***, P < 0.001; ****, P < 0.0001. [file JEV2-15-e70266-s003.tif]

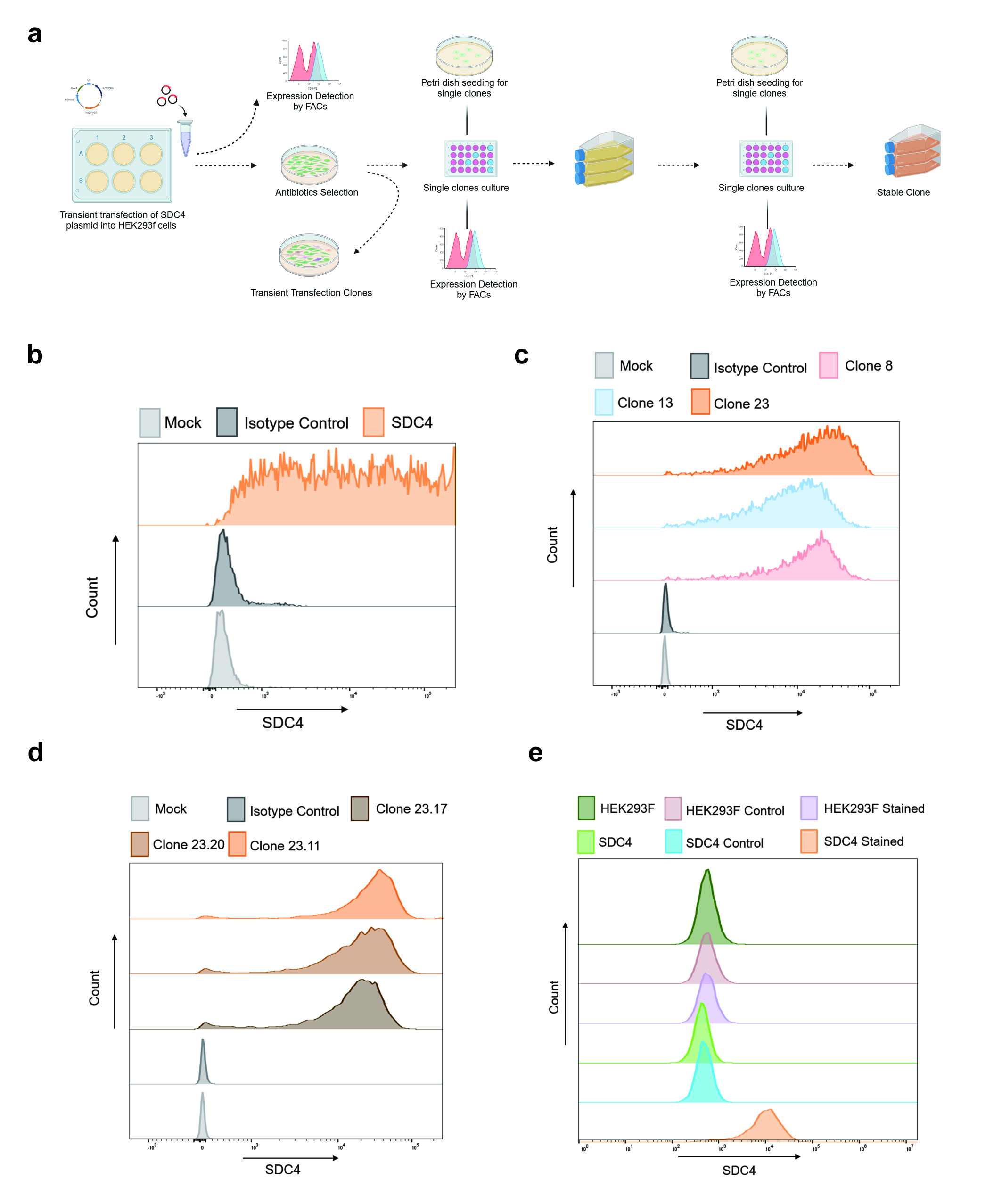

Supplement: Supplementary file 3 — Supplementary Figure 3. The process of establishing an SDC4 expressing HEK293F clone. a) Experimental design for the transfection, which included the production of transient transfection clones and the selection of stable clones. b) Flow cytometry data of SDC4 expression on the transient transfection clones. c) Flow cytometry data of three clones after the first selection process. d) Flow cytometry data of the top three clones after the second selection process. e) Flow cytometry data comparing SDC4 expression of HEK293F wild‐type cells and final selected SDC4 HEK293F clone. [file JEV2-15-e70266-s005.tif]

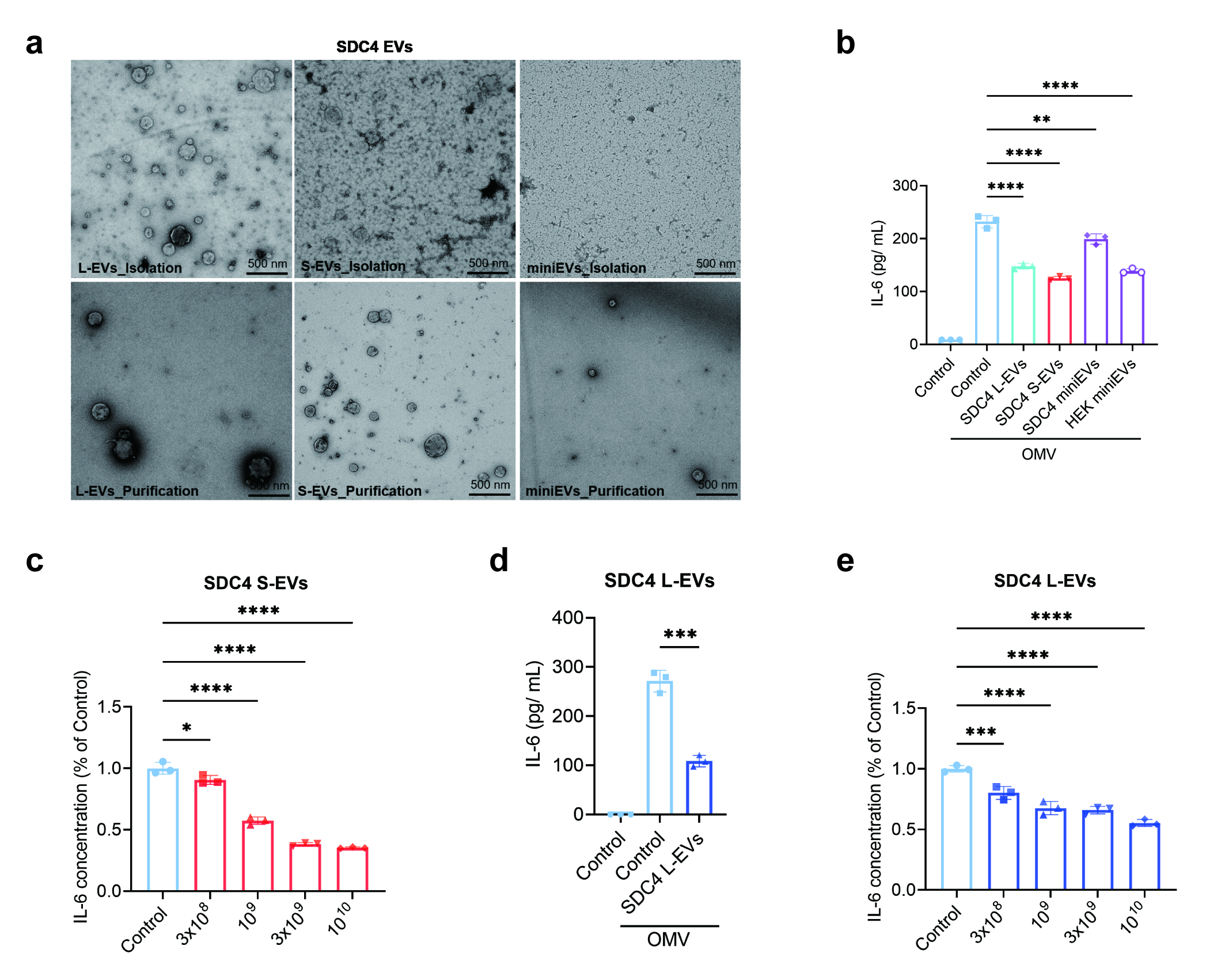

Supplement: Supplementary file 4 — Supplementary Figure 4. Characterization and anti‐inflammation function of L‐EVs, S‐EVs and miniEVs of SDC4 overexpressing HEK293F cells. a) TEM images of SDC4 L‐EVs, S‐EVs, and miniEVs after ultracentrifugation (called “isolation”—upper panels) and after iodixanol density gradient (called “purification”—lower panels). b) IL‐6 concentration in the supernatant of RAW 264.7 cells after exposure to OMV (100 ng/mL) followed by treatment with EVs (109 /mL). c) IL‐6 concentration in the supernatant of RAW 264.7 cells after exposure to OMV (100 ng/mL) followed by treatment of increasing concentrations of SDC4 S‐EVs. d) IL‐6 concentration in the supernatant of RAW 264.7 cells after exposure to OMV (100 ng/mL) followed by treatment with SDC4 L‐EVs _ isolation (109 /mL). e) IL‐6 concentration of the supernatant of RAW264.7 cells after exposure to OMV (100 ng/mL) followed by treatment of increasing concentrations of SDC4 L‐EVs _ isolation. Data were analyzed using one‐way ANOVA. *, P <0.05; **, P < 0.01; ***, P < 0.001; ****, P < 0.0001. [file JEV2-15-e70266-s004.tif]

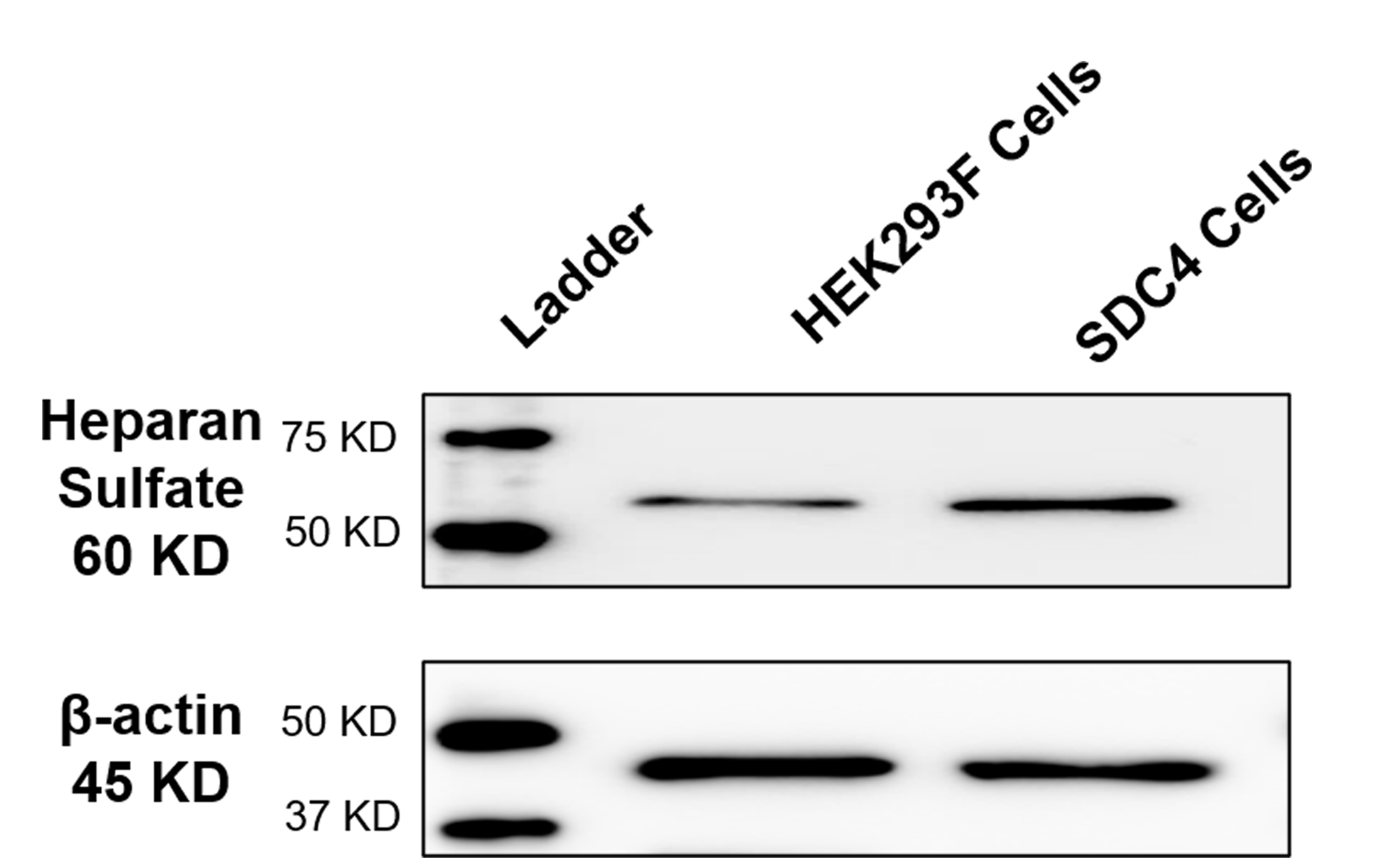

Supplement: Supplementary file 5 — Supplementary Figure 5. Western blot of heparan sulfate expression of wild‐type HEK293F cells vs SDC4 overexpressing HEK293F cells. [file JEV2-15-e70266-s001.tif]
